# Neural crest stem cells from human epidermis of aged donors maintain their multipotency *in vitro* and *in vivo*

**DOI:** 10.1038/s41598-019-46140-9

**Published:** 2019-07-05

**Authors:** Samaneh Moghadasi Boroujeni, Alison Koontz, Georgios Tseropoulos, Laura Kerosuo, Pihu Mehrotra, Vivek K. Bajpai, Surya Rajan Selvam, Pedro Lei, Marianne E. Bronner, Stelios T. Andreadis

**Affiliations:** 1 Department of Chemical and Biological Engineering, University at Buffalo, Buffalo, NY 14260 USA; 2 Division of Biology and Biological Engineering, California Institute of Technology, Pasadena, CA 91126 USA; 3 NIH/NIDCR, Bethesda, MD 20892 USA; 4 Chemical & Systems Biology, Stanford School of Medicine, Palo Alto, CA 94305 USA; 5 Department of Psychological and Brain Sciences, University of Louisville, Kentucky, KY 40292 USA; 6 Department of Biomedical Engineering, University at Buffalo, Buffalo, NY 14260 USA; 7 Center of Excellence in Bioinformatics and Life Sciences, Buffalo, NY 14263 USA

**Keywords:** Adult stem cells, Multipotent stem cells

## Abstract

Neural crest (NC) cells are multipotent stem cells that arise from the embryonic ectoderm, delaminate from the neural tube in early vertebrate development and migrate throughout the developing embryo, where they differentiate into various cell lineages. Here we show that multipotent and functional NC cells can be derived by induction with a growth factor cocktail containing FGF2 and IGF1 from cultures of human inter-follicular keratinocytes (KC) isolated from elderly donors. Adult NC cells exhibited longer doubling times as compared to neonatal NC cells, but showed limited signs of cellular senescence despite the advanced age of the donors and exhibited significantly younger epigenetic age as compared to KC. They also maintained their multipotency, as evidenced by their ability to differentiate into all NC-specific lineages including neurons, Schwann cells, melanocytes, and smooth muscle cells (SMC). Notably, upon implantation into chick embryos, adult NC cells behaved similar to their embryonic counterparts, migrated along stereotypical pathways and contributed to multiple NC derivatives *in ovo*. These results suggest that KC-derived NC cells may provide an easily accessible, autologous source of stem cells that can be used for treatment of neurodegenerative diseases or as a model system for studying disease pathophysiology and drug development.

## Introduction

The neural crest (NC) is a stem cell population, unique to vertebrates, that arises in vertebrate embryos during nervous system formation^1^. From their site of origin in the forming central nervous system, neural crest stem cells delaminate and migrate throughout the peripheral system of the developing embryo. Subsequently, they differentiate into various cell types including peripheral neurons, glia, cardiac outflow tract smooth muscle cells, melanocytes, adipocytes as well as chondrocytes and osteoblasts of the craniofacial skeleton^2^. Therefore, they represent a cell population with great potential for use in cell replacement for regenerative medicine.

*In vitro*, NC cells have been derived from embryonic stem cells and induced pluripotent stem cells^3,4^, as well as with direct reprogramming from fibroblasts by introduction of transcription factors, such as Sox10^5^ or FOXD3^6^. Interestingly, NC cells have also been isolated from various adult tissues including dorsal root ganglia, gut, heart, olfactory sheath, hair follicles and craniofacial tissue^2,7–9^. These cells maintain their multipotency as they can be coaxed to differentiate into neuronal and glial cells^10–17^, smooth muscle cells^10,12,14^, melanocytes^14,18^, bone cells^18–23^, adipocytes^10,18–20^, and chondrocytes^10,14,18^. As a result, several groups have proposed their use in applications including treatment for spinal cord injury^24^, deafness^25^, ocular repair^26–28^ or periodontal regeneration^29^. However, clinical application is hampered by the need for genetic modification in reprogramming or the limited accessibility of adult tissues where they reside.

Recently, we showed that NC cells can be derived from neonatal keratinocytes of the interfollicular epidermis, without introduction of transcription factors or reprogramming to pluripotency^30^. However, it was not clear whether multipotent and functional NC cells can be derived from the adult epidermis of aged donors, who have the greatest need for cell therapies. Here we show that adult NC cells from elderly donors can also be obtained from epidermal cultures by treatment with a growth factor cocktail containing FGF2 and IGF1. Adult NC cells derived from KC cultures (KC-NC) from different donors expressed key NC markers including transcription factors SOX10, FOXD3, PAX3 and intermediate filament protein, NES. Surprisingly, NC cells from aged epidermal tissues showed limited signs of cellular senescence and younger epigenetic age than epidermal KC. They also maintained their multipotency as evidenced by differentiation into all NC-specific lineages including neurons, Schwann cells, melanocytes, and smooth muscle cells (SMC). Most notably, lineage tracing experiments by implantation into chick embryos showed that KC-NC from aged donors could migrate along stereotypical pathways and differentiate into multiple NC derivatives in ovo, including neurons, glia, SMC and putative melanoblasts. Given the multipotency of KC-NC and the accessibility of human epidermis, our results suggest that KC-NC have great potential as an autologous cell source for regenerative medicine and stem cell biology.

## Materials and Methods

### Isolation of epidermal cells

Skin from the right thigh of human cadavers ranging from 67 to 93 years of age was obtained from Gross Anatomy Lab of the University at Buffalo in accordance with appropriate guidelines and regulations. Written informed consent was provided before death by the donors who donated their bodies to the University at Buffalo for teaching purposes, scientific research or such purposes as the University, or its authorized representatives, shall in their sole discretion deem advisable. The UB Institutional Review Board (IRB) determined that researchers using any materials from those donors do not need to get specific permission as UB already has blanket permission to use them as needed. The skin of the donors was harvested as it became available and the total number of donors used in this study was n = 11 (10 male and 1 female). After washing three times with phosphate-buffered saline (PBS), the skin tissues were dissected into small pieces (~1 cm × 1 cm) and enzymatically digested using dispase II protease (Sigma, St. Louis, MO) for 15–20 hr at 4 °C. The epidermis was separated from the dermis manually using fine forceps and then treated with trypsin-EDTA (0.25%) (Life Technologies, Carlsbad, CA) for about 10–15 min at 37 °C. After filtering through a 70 μm cell strainer (BD Biosciences, Franklin Lakes, NJ), the cell suspension was centrifuged and resuspended in keratinocyte growth medium (KCM) containing 3:1 mixture of DMEM (high glucose) and Ham’s F-12 medium (Life Technologies) supplemented with 10% (v/v) fetal bovine serum (FBS, Atlanta Biologicals, Flowery Branch, GA), 100 nM cholera toxin (Vibrio Cholerae, Type Inaba 569 B, Millipore, Burlington, MA), 5 μg/ml transferrin (Life Technologies), 0.4 μg/ml hydrocortisone (Sigma), 0.13 U/ml insulin (Sigma), 1.4 × 10^−4^ M adenine (Sigma), 2 × 10^−9^ M triiodo-L-thyronine (Sigma), 1x antibiotic-antimycotic (Life Technologies) and 10 ng/ml epidermal growth factor (EGF, added 3 days post-seeding, BD Biosciences). The resuspension was then cultured on a monolayer of growth arrested 3T3-J2 mouse fibroblast feeder cells. The harvested cells that were cultured in KCM formed colonies in 8 –10 days and the feeder cells were removed using versene treatment for about 10 min at 37 ^o^C. The remaining cells were treated with trypsin-EDTA (0.25%); the trypsin was neutralized with PBS containing 10% FBS and the cells were cultured on collagen type I coated tissue culture plates (10 μg collagen type I per cm^2^; BD Biosciences) in keratinocyte serum free growth medium (KSFM, Epilife medium with Human Keratinocyte Growth Supplement, Life Technologies) until NC induction. KC were used immediately after isolation (passage 1) for all experiments. Neonatal cells were isolated from glabrous neonatal (1- to 3-day-old neonates) foreskin tissues that were obtained from the John R. Oishei Children’s Hospital, Buffalo, NY according to IRB of John R. Oishei Children’s Hospital. Samples were regularly discarded tissues from foreskin circumcisions. Since there was not any identifying data from patients, an exemption for obtaining patient consent was granted by IRB of John R. Oishei Children’s Hospital. All protocols were in accordance with appropriate guidelines and regulations.

### Induction of neural crest stem cells

To obtain NC cells, KC were cultured at a density of 3–5 × 10^3^ cells/cm^2^ on collagen I coated tissue culture plates and exposed to Neural Crest Induction Medium (NCIM) containing basal medium (EBM2 medium; Lonza, Basel, Switzerland) supplemented with 2% (v/v) FBS, 10 µg/ml heparin (Lonza), 100 µg/ml ascorbic acid (Lonza), 0.5 µg/ml hydrocortisone, 1x Gentamicin/Amphotericin-B (Lonza), 10 ng/ml fibroblast growth factor 2 (FGF2, Isokine, Iceland), and 10 ng/ml Insulin like growth factor 1 (IGF1, Lonza). After 2–3 days of induction, NC cells could be seen surrounding KC colonies and by day 10 they had proliferated extensively occupying almost the areas between KC colonies. At that time, NC cells were separated from KC by differential trypsinization for about 3 min and re-plated for further experiments. NC cells were derived from all donors (n = 11) and each assay as described below was conducted with cells from at least n = 3 donors.

### Immunostaining and fluorescence microscopy

After washing with PBS, the cells were fixed with 4% (v/v) paraformaldehyde (10 min, room temperature (RT); Sigma), permeabilized using 0.1% (v/v) triton X-100 (Sigma) for 10 min at RT, washed 3 times with PBS and blocked with 0.01% (v/v) triton X-100 and 5% (v/v) normal goat serum (Life Technologies) in PBS. Then cells were incubated with primary antibodies overnight at 4 °C, followed by 1 hr incubation with secondary antibody (Alexa 488- or Alexa 594-conjuated anti-IgG antibody, Thermo Fisher Scientific, Grand Island, NY, 1:200 dilution) at RT and counterstained with Hoechst 33342 (Thermo Fisher Scientific) for 5 min at RT. Cells incubated with only secondary antibody served as negative controls. Images were taken using a Zeiss Axio Observer Z1 inverted microscope with an ORCA-ER CCD camera (Hamamatsu, Japan). The images were captured using fixed exposure time for each fluorescent dye for all samples. Fluorescence intensity and cells numbers were quantified using NIH ImageJ.

### Mitochondrial membrane potential

MitoTracker Red CMXRos (Thermo Fisher Scientific) was used per manufacturer’s instructions in order to assess mitochondrial membrane potential. Briefly, cells were incubated for 10 min in staining solution containing 100 μM MitoTracker® probe, washed 3 times with PBS and counterstained with the nuclear dye Hoechst 33342.

### Determination of ROS

Mitochondrial superoxide was assessed using the MitoSOX™ Red Mitochondrial Superoxide Indicator (Thermo Fisher Scientific) per manufacturer’s instructions. Briefly, cells were incubated for 10 min with 5 μM MitoSOX™ reagent working solution, washed gently 3 times with PBS and counterstained with the nuclear dye Hoechst 33342.

### DNA methylation age of KC and NC cells

KC were isolated from 3 different neonatal and 3 different adult donors. After isolation, KC were passaged in KSFM and then treated with NCIM for 9–11 days to obtain NC cells. Human cell samples were collected and stored in DNA/RNA Shield™ buffer (www.zymoresearch.com–Cat. No. R1150, Zymo Research, Irvince, CA). Genomic DNA was purified from cells using the Quick-DNA™ Miniprep Plus Kit (Cat. No. D4068, Zymo Research) and quality controls were done by Nanodrop. Bisulfite conversion was performed using the EZ DNA Methylation-Lightning™ Kit (Cat. No. D5030, Zymo Research) according to the standard protocol. Samples were then enriched for sequencing of >500 age-associated gene loci. DNA methylation values of KC and NC stem cell samples were obtained from the sequence data and used to assess DNA age according to Zymo Research’s proprietary DNAge® predictor.

### Gel compaction assay and vasoreactivity assay for testing adult NC-derived smooth muscle cell function

To measure the function of NC-derived SMC, we employed two assays as published previously: the gel compaction and the vascular contractility assays^31,32^. For the gel compaction assay, NC-derived SMC or human aortic SMC (ASMC; Life Technologies) were mixed with 0.8 ml of fibrinogen (Enzyme Research Laboratories, South Bend, IN). The mixture then was polymerized using 0.2 ml of thrombin (Sigma) in a 24-well plate at 37 °C for 1 hour. The final concentration of fibrinogen and thrombin in the fibrin hydrogel was 2.5 mg/ml and 2.5 U/ml, respectively. After 1 hr, the fibrin gels were detached from the walls using a fine needle, enabling the gel to compact. Images were taken at the indicated times (0, 24, 48, 72, and 96 hr) by ChemiDoc Imaging system (BioRad, Hercules, CA) and the area of each hydrogel (A) was measured over time using ImageJ and normalized to the initial hydrogel area (A0).

The vascular contractility, also known as vasoreactivity assay was performed as described previously^31,32^. Briefly, fibrin hydrogels containing NC cells, NC-derived SMC, or control human aortic SMC (HASMC, 10^6^ cells/ml) were polymerized around a 6-mm diameter mandrel of poly (di-methyl siloxane) to form cylindrical constructs. After 1 hr of incubation, hydrogels were detached from the walls and incubated in Tissue Engineered Vessel (TEV) medium containing DMEM and 10% FBS supplemented with 2 μg/ml insulin (Sigma), 2 ng/ml TGF-β1 (Biolegend, San Diego, CA), 300 μM ascorbic acid phosphate (Alfa Aesar, Tewksbury, MA), and 2 mg/ml e-amino-n-caproic acid (Sigma) for 2 weeks. After 2 weeks, tissues were released from the mandrels and mounted on two hooks through the lumen in tissue bath containing Krebs–Ringer solution at 37 °C. Hydrogels were equilibrated at a basal tension of 1.0 g and constant length for approximately 45–60 min. After equilibration, isometric contractions were recorded in response to vascular agonists: (endothelin-1, 20 nM), the thromboxane A2 mimetic U46619 (1 μM) or potassium chloride (KCl, 118 mM). Relaxation of maximally contracted tissue constructs was measured in response of Y27632 (10 mM) using a PowerLab data acquisition unit and analyzed with Chart5 software (ADInstruments, Colorado Springs, CO).

### Differentiation of adult NC cells into NC derivatives

#### Schwann cell differentiation

Adult NC cells were plated on poly-L-ornithine/laminin coated plates and cultured in Schwann cell (SC) differentiation medium containing EBM2 as basal medium, 2% (v/v) FBS, 100 ng/ml ciliary neurotrophic factor (Life Technologies), 100 ng/ml NRG1, 4 ng/ml FGF2, 200 mg/ml ascorbic acid, 0.5× Glutamax (ThermoFisher Scientific), and 10 μM SB431542 (Sigma) for 5 weeks.

#### Melanocyte differentiation

Adult NC cells were cultured in EBM2 basal medium supplemented with 5% FBS, SCF (100 ng/ml), endothelin-3 (200 nM), WNT1 (50 ng/ml), FGF2 (10 ng/ml), insulin (5 μg/ml), cholera toxin (1 pM), 12-O-tetra-decanoylphorbol-13-acetate (TPA, 10 nM; Sigma) and SB431542 (10 μM) for five weeks. At that time, we examined melanin secretion, using the *L-*DOPA assay, for which adult NC-Mel were fixed with 4% (w/v) paraformaldehyde for 20 min at room temperature. After washing three times with PBS, the cells were incubated with freshly prepared 5 mM L-DOPA (Sigma) overnight at 37 °C, fixed with 4% (v/v) paraformaldehyde for 20 min at RT, washed with PBS and visualized using bright field microscopy.

#### Smooth muscle cell (SMC) differentiation

KC-NC were induced to SMC in DMEM plus 10% (v/v) FBS and 10 ng/ml TGF-β1 for two weeks. ASMC were used as the positive control.

#### Peripheral neuron differentiation

Adult NC cells were cultured on poly-L ornithine (100 ng/ml; Sigma)/laminin (10 mg/ml; EMD Millipore, Billerica, MA) coated dishes and exposed to neuron differentiation media containing Neurobasal plus medium (Thermo Fisher Scientific) with BMP2 (10 ng/ml; R&D systems, Minneapolis, MN), SB431542 (10 μM), B27 plus (Thermo Fisher Scientific), N2 supplement (R&D systems), Brain-derived neurotrophic factor (BDNF; 10 ng/ml; Thermo Fisher Scientific), Glial cell-derived neurotrophic factor (GDNF;10 ng/ml; Sigma), Nerve growth factor (NGF; 10 ng/ml; R & D systems), Neurotrophin 3 (NT3; 10 ng/ml; Sigma), ascorbic acid (200 μM; Sigma) and cyclic adenosine monophosphate (0.5 mM cAMP; Sigma), CHIR 99021 (0.5 μM, only on day 1; Sigma), 2% FBS (from day 1–5), IWP-4 (100 nM days 4–6; 1 μM thereafter; Tocris Bioscience, Minneapolis, MN).

### *In ovo* transplantation

Adult NC stem cells were transduced with lentivirus containing CMV promoter driving expression of the ZsGreen+ reporter. About 50–60% of cells were ZsGreen+ as evidenced by fluorescence microscopy. KC-NC or control KC were dissociated using 2 mL of Accuprime (#AM-105, Innovative Cell Technologies Inc., San Diego, CA) and incubated at 37 °C for 5 minutes. The cells were washed twice with 1 mL of Ringer’s balanced salt solution, and spun down for 7 minutes at 200 G, resuspended into 10 to 20 µL of cell medium, and loaded into a thin pulled glass needle pipette. The cells were injected into the migratory cranial NC stream of Hamburger-Hamilton Stage 9–12 chick embryos. In total, 157 embryos were successfully injected with experimentally induced NC cells, and 55 with control cells. Embryos were examined for visible GFP fluorescence under a Leica fluorescent microscope to determine the efficiency of injections, covered with sterile surgical tape, and incubated at 37 °C. After 48–72 hours, the surviving embryos were dissected out, fixed with 4% paraformaldehyde in PBS overnight at 4 °C and washed 3 times with PBS. Thirty-nine experimental embryos (25% survival rate) and 19 control embryos (35% survival rate) survived and were processed.

Fixed embryos were embedded in gelatin and sectioned transversely at 14 µm on a cryostat. Sections were examined under a fluorescent Apotome microscope (Zeiss Axioscope 2 and Zeiss ApoTome.2) for GFP signal. Sections containing GFP positive cells were blocked with a 2.5% goat and 2.5% donkey serum solution in PBS-Tween 0.2%, and antibodies were added to the same blocking solution. Immunostaining was performed with the following antibodies: for glia, BLBP (ABN14, EMD Millipore, 1:200, antigen retrieval was performed by placing slides in sodium citrate buffer, pH 6, in a 68 °C water bath overnight, prior to blocking); for neurons HuC/D (Invitrogen/molecular probes 16A11 1:100); for smooth muscle, αSMA (Sigma A5228 1:2000); for nuclei, DAPI (1:1000). Secondary Alexa dye-conjugated antibodies (Molecular Probes) were used at 1:1000. Slides were imaged using fluorescence microscopy (Zeiss Axioscope 2 and Zeiss ApoTome.2).

## Results

### Adult neural crest stem cells derived from keratinocyte cultures

**Figure 1:**
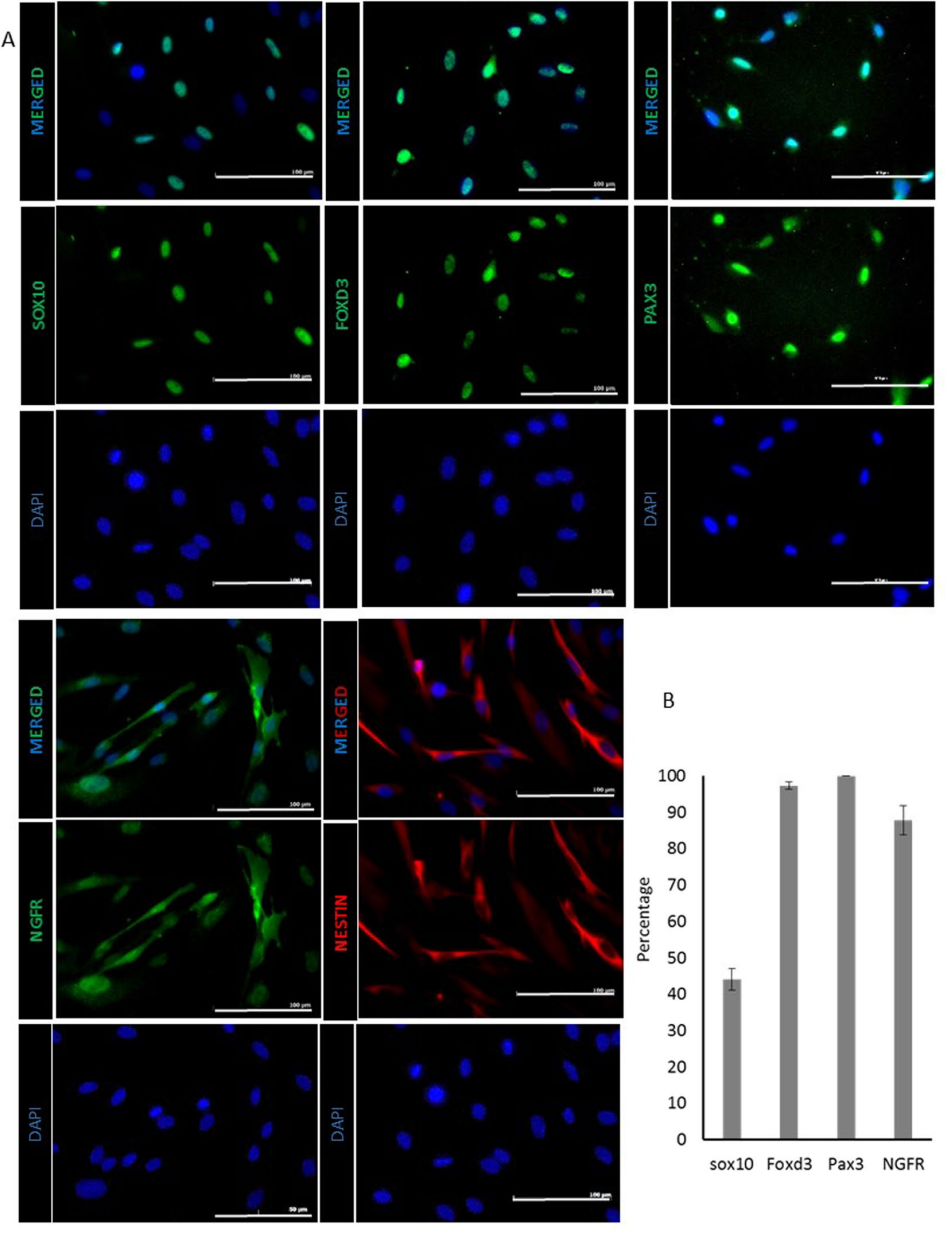
Adult NC cells derived from keratinocyte cultures express NC specific markers. **(A)** Immunostaining of adult NC cells for SOX10, FOXD3, PAX3, NGFR and NESTIN. Scale bar is 100 μM. **(B)** Percentage of adult NC cells expressing SOX10, FOXD3, PAX3, and NGFR after two weeks of culture. All values are mean ± SD. Each experiment was repeated three times.

Previously we showed that neural crest stem (NC) cells can be isolated from the interfollicular epidermis of glabrous skin from 1–3 day old neonates. However, it was not clear that NC-like cells can also be derived from adult epidermis. To this end, we derived NC cells from epidermal KC of human skin tissues of adult donors ranging from 67 to 93 years of age (n = 11 donors). KC were initially cultured in calcium free medium (KSFM). When the medium was changed to the NC induction medium (NCIM consisted of EBM2 basal medium containing FGF2, IGF1, ascorbic acid, hydrocortisone, heparin, and 2% FBS), KC formed colonies that were surrounded by a number of small, spindle shaped cells 5–6 days later. Immunostaining showed that these cells expressed key epidermal NC markers including lineage-specific transcription factors such as SOX10, FOXD3, PAX3, the NGF receptor (NGFR) and the intermediate filament protein, NES (Fig. 1A). Almost all cells expressed NES; the vast majority expressed Pax3 (92.68 ± 6.75%), FoxD3 (97.3 ± 0.99%) and NGFR (87.7 ± 4.01%), while about 40.0 ± 2.96% of cells were positive for Sox10 after 14 days in NCIM (4 fields of view containing n ≥ 500 cells) (Fig. 1B).

### Aging hallmarks in adult NC cells. 

**Figure 2:**
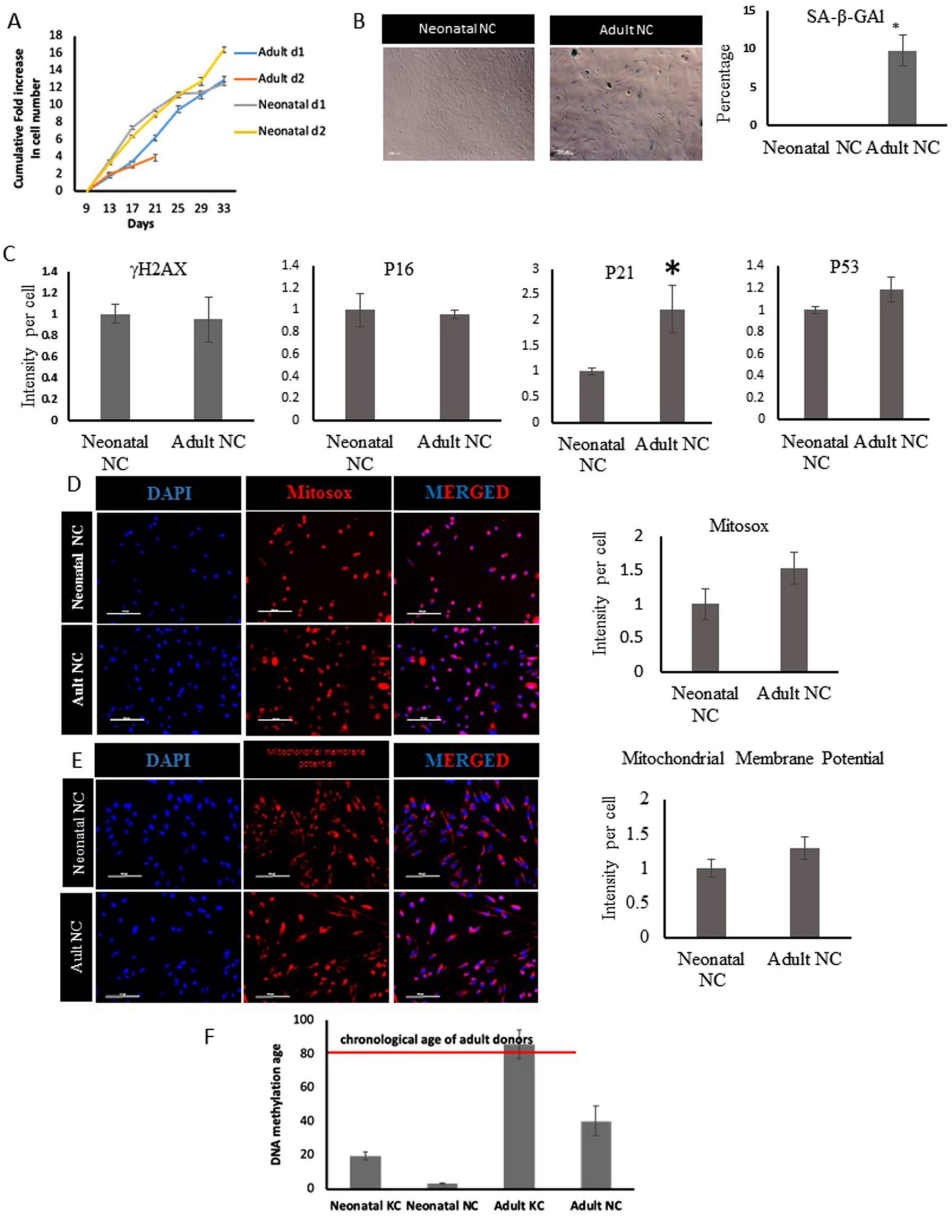
Aging hallmarks in NC derived from adult and neonatal KC. **(A)** Proliferation kinetics. **(B)** SA-β-Gal staining and quantification. **(C)** Quantification of the staining intensity of γH2AX, P16, P21, and P53 normalized to the intensity of neonatal NC cells. **(D)** Total reactive oxygen species (ROS) in adult vs. neonatal NC cells evaluated using the MITOSOX reagent. **(E)** Mitochondrial membrane potential of adult vs. neonatal NC cells. Scale bar is 100 μm. **(F)** DNA methylation age of adult and neonatal KC and KC-NC. All values are mean±SD. Each experiment was repeated three times.

Next, we examined the aging hallmarks in NC cells from neonatal vs. adult donors that were cultured only for one passage, in order to capture differences due to donor age rather than replicative senescence. NC cells from aged donors could be maintained in culture for at least 3 passages with an average doubling time of 49.45 ± 1.73 hr (n = 2 donors) (Fig. 2A), which was significantly longer than that of neonatal NC cells (28.45 ± 1.1 hr, n = 2 donors, p < 0.05). In addition, a small percentage of adult NC cells expressed senescence-associated-β-galactosidase (SA-β-Gal, neonatal: 0% vs. adult: 9.81 ± 2.01%, p < 0.0001, n ≥ 600 cells) (Fig. 2B).

Interestingly, immunostaining did not show significant differences between the neonatal and adult NC cells with respect to the DNA damage marker γH2AX (fluorescence intensity (FI) per cell for neonatal:1.0 ± 0.08 vs. adult: 0.94 ± 0.21, p > 0.05, n ≥ 400 cells) (Fig. 2C and Fig. S1) or expression of senescence associated markers p16 or p53 (p16, FI for neonatal: 1 ± 0.14 vs. adult = 0.95 ± 0.03, p = 0.6381; p53, neonatal: 1 ± 0.03 vs. adult: 1.18 ± 0.11, p = 0.1120, n = 3 donors) but they showed upregulation of p21 (p21: FI for neonatal: 1 ± 0.06 vs. adult: 2.21 ± 0.46, p = 0.039, n = 3 donors) (Fig. 2C and Fig. S1). Finally, adult NC cells showed similar levels of superoxide (MitoSox, FI for neonatal: 1 ± 0.22 vs. adult: 1.52 ± 0.241, p = 0.534, n = 3 donors) (Fig. 2D) and mitochondrial membrane potential (FI for neonatal: 1 ± 0.13 vs. adult: 1.28 ± 0.15, p = 0.1096) (Fig. 2E) as compared to neonatal NC.

### Epigenetic age of adult KC and KC-derived NC

Recent publications have shown chronological age has a profound effect on genome-wide DNA methylation levels and that methylation of CpG dinucleotides can be used to predict the epigenetic age or else known as DNA methylation-based (DNAm) age of human tissues and cells using linear regression algorithms or epigenetic “age estimators” such as the Horvath clock^33–35^. Since we did not observe significant differences in several aging hallmarks between adult and neonatal KC-NC despite the large difference in the chronological age of the donors, we employed this method to determine the DNAm age of KC and KC-NC from neonatal and adult donors. Principle component analysis (PCA) of the differentially methylated CpG regions in KC and KC-NC from 3 neonatal and 3 adult donors showed clustering into four distinct groups (Fig. S2A), indicating differences between cell types but also between different age donors. Moreover, a heat map and associated dendrogram showed that although there were differences between the methylation profiles of adult and neonatal cells, the biggest differences were seen between the two cell types, KC and KC-NC (Fig. S2B). Finally, linear regression calculations based on the Horvath clock algorithm showed that the DNAm age of adult KC (85.96 ± 8.47 years; n = 3 donors) was similar to the chronological age of adult donors (80.33 ± 9.97 years; p = 0.497, n = 3 donors;). Surprisingly, the predicted DNAm age of adult KC-NC (40.33 ± 9.19 years; n = 3 donors) was significantly younger than the chronological age of the donors (80.33 ± 9.97 years; p = 0.007, n = 3 donors), suggesting that NC cells may be aging much more slowly than KC (Fig. 2F). These results suggested that NC cells may retain their multipotency despite the late age of the donors.

### Differentiation of adult NC cells to functional neural crest derivatives

To address this hypothesis, we examined the propensity of adult KC-NC to differentiate into NC derivatives, including Schwann cells, neurons, melanocytes and smooth muscle cells.

#### Schwann cells

**Figure 3:**
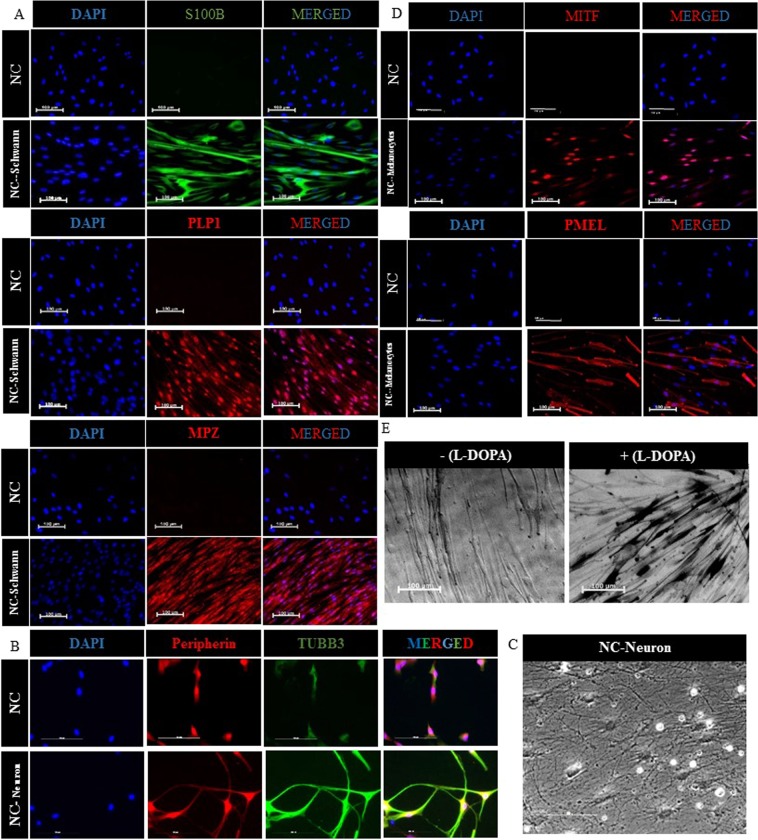
Differentiation of adult KC-NC to functional neural crest derivatives. Immunostaining for (**A**)Schwann cell specific markers including. S100B, PLP1, and MPZ, and **(B)** peripheral neuron specific markers, Peripherin and TUBB3. **(C)** Phase image shows the morphology of adult NC-derived neurons. **(D)** Melanocyte specific markers MITF and PMEL. Scale bar is 100 μm. **(E)** L-DOPA assay showing melanin secretion indicating tyrosinase activity.

NC cells differentiated into Schwann cells in the presence of differentiation medium containing EBM2 basal medium supplemented with 2% FBS, 100 ng/ml CNTF, 100 ng/ml NRG1, 4 ng/ml FGF2, 200 µg/ml ascorbic acid and 0.5x Glutamax, for 5 weeks. Immunostaining showed that almost all the cells were positive for S100B, PLP1, and MPZ (Fig. 3A).

#### Peripheral neurons

NC cells differentiated into peripheral neurons using neuron differentiation medium (Neurobasal plus media with BMP2 (10 ng/ml), SB431542 (10 μM), B27 plus, N2 supplement, Brain-derived neurotrophic factor (BDNF; 10 ng/ml), Glial cell-derived neurotrophic factor (GDNF; 10 ng/ml), Nerve growth factor (NGF; 10 ng/ml), Neurotrophin 3 (NT3; 10 ng/ml), Ascorbic acid (200 μM) and cyclic adenosine monophosphate (0.5 mM cAMP), CHIR 99021 (0.5 μM, only on day 1), 2% FBS (from day 1–5), IWP4 (100 nM days 4–6; 1 μM thereafter). After 14 days in differentiation medium, the cells developed long processes and expressed typical neuronal markers such as Peripherin (83.85 ± 0.3%, n = 567 cells) and TUBB3 (59.48 ± 0.2%, n = 1,832 cells) (Fig. 3B,C).

#### Melanocytes

For melanocyte differentiation, NC cells were cultured in EBM2 basal medium supplemented with 5% FBS, SCF (100 ng/ml), endothelin-3 (200 nM), WNT1 (50 ng/ml), FGF2 (10 ng/ml), insulin (5μg/ml), cholera toxin (1 pM), 12-O-tetra-decanoylphorbol-13-acetate (TPA, 10 nM) and SB431542 (10 μM). After 5 weeks of differentiation, 52 ± 6.33% of the cells expressed the melanocyte-specific transcription factor, MITF and 40 ± 6.61% expressed the pre-melanosome transmembrane glycoprotein, PMEL (n = 780 cells) (Fig. 3D). Notably, NC-derived melanocytes produced melanin, clearly indicating tyrosinase activity–specific for melanocytes –as evidenced by the L-DOPA assay (Fig. 3E).

#### Smooth muscle cells

**Figure 4:**
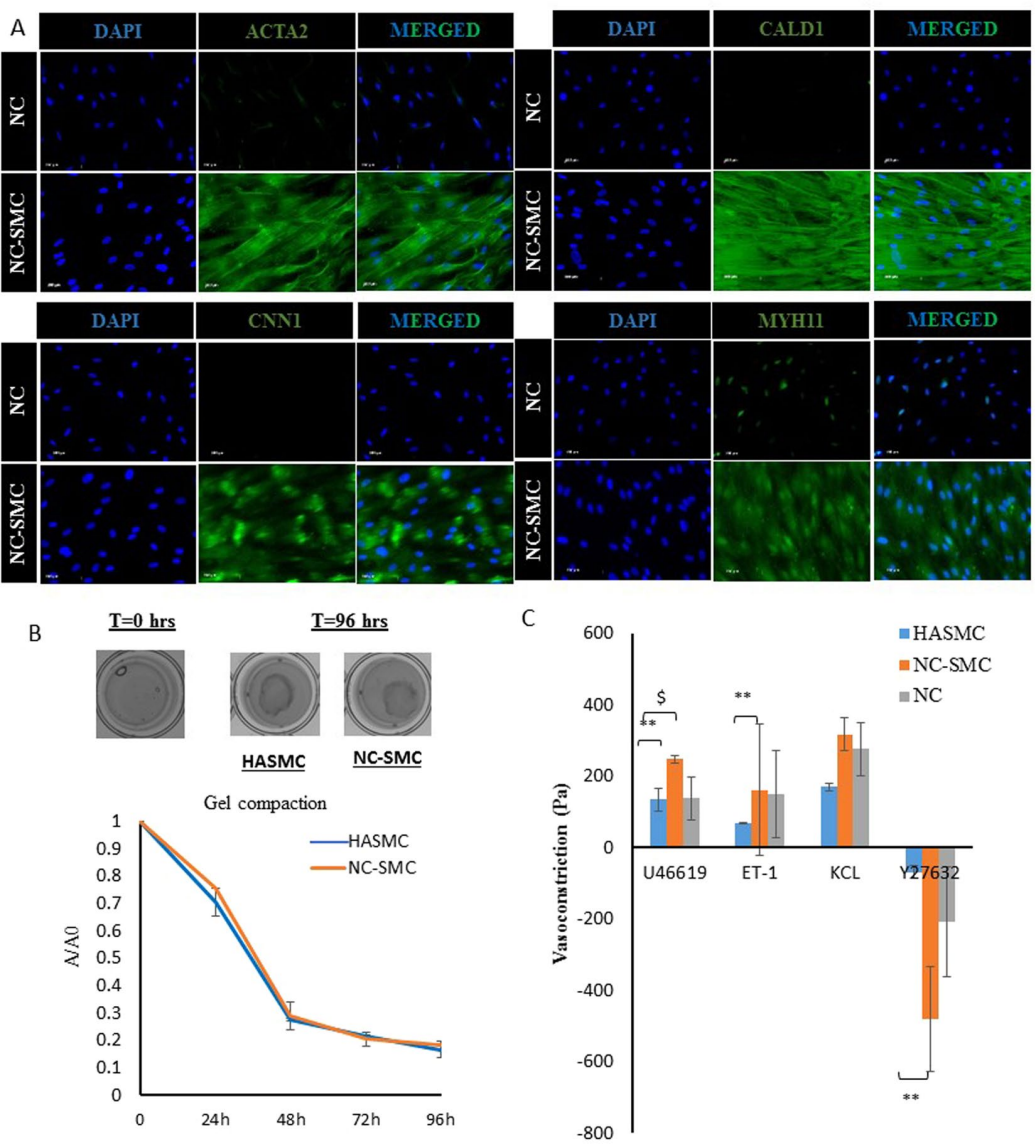
Differentiation of adult KC-NC to functional SMC. (**A**) Immunostaining of SMC specific markers including ACTA2, CALD1, CNN1, and MYH11. **(B)** Hydrogel compaction of HASMC and NC-SMC. **(C)** Vasoreactivity of HASMC, NC-SMC, and NC cells based bioengineered vascular constructs in response to U46619, Endothelin-1, KCl, and Y27632. All values are mean ± SD of triplicates. Each experiment was repeated three times.

NC cells were coaxed to differentiate into SMC in DMEM supplemented with 10μg/ml TGF-β1 for two weeks. Almost all of the NC-SMC showed positive staining for ACTA2, CALD1 and MYH11, and about 92.85 ± 8.5% (n = 308 cells) expressed CNN1 (Fig. 4A).

In addition, we examined the contractile function of NC-SMC using two well-established assays: hydrogel compaction and contractility (or vasoreactivity). For hydrogel compaction, cells were embedded in fibrin hydrogels that were allowed to compact over time. As shown in Fig. 3B, NC-SMC compacted fibrin hydrogels over time down to less than 20% of their initial area (96 hr), which was very similar to compaction by human aortic (hA)SMC (Fig. 4B).

For vasoreactivity, we prepared cylindrical tissue equivalents by embedding cells (NC cells, NC-SMC or HASMC) in fibrin hydrogels that were polymerized around cylindrical mandrels, as we published before^31,36,37^. After two weeks in culture in the presence of TGF-β1, the cells compacted the hydrogels down to ~5% of their original volume yielding cylindrical constructs with wall thickness of less than 500 µm. At that time, rings of tissue constructs were placed in isolated tissue baths to measure isometric tension generated in response to several vascular agonists: the thromboxane A2 mimetic U46619, Endothelin-1, and KCl as well as the vasodilator, Y27632 (Fig. 4C). Reactivity of NC-SMC based tissue constructs was comparable (KCl, p = 0.2730, n = 3) or superior (U46619, p < 0.01, n = 3; ET-1, p < 0.05, n = 3; or Y27632, p < 0.05, n = 3) to that of hASMC based tissues. Interestingly, NC cells differentiated into functional SMC in 3D, although differentiating NC cells into SMC (NC-SMC) prior to generating the 3D constructs resulted in even higher contractility in response to U46619 (p < 0.05, n = 3). These results indicate that functional adult KC-NC differentiated into functional, contractile SMC.

### NC cells migrate and differentiate into NC lineages in ovo

**Figure 5:**
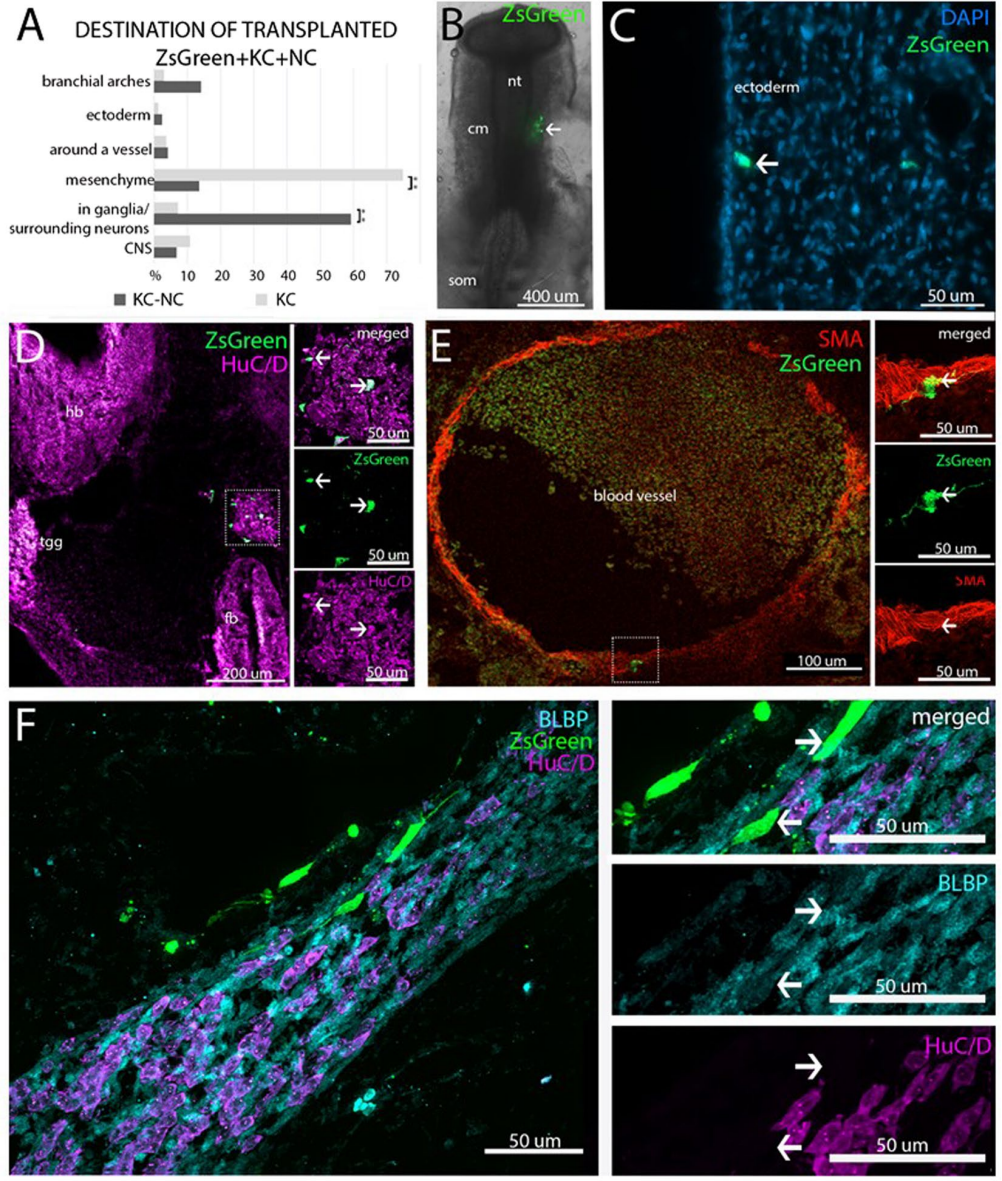
Adult KC-NC migrate to neural crest sites and differentiate into appropriate derivatives in ovo. **(A)** Summary of locations in which ZsGreen positive transplanted cells were found in 3–4-day-old chicken embryos in representative embryos. Percentage of experimental transplanted cells detected in each target structure in the developing chick embryos (n = 8 embryos; total number of detected ZsGreen+ cells = 382 out of ~2000 transplanted cells) compared with the percentage of control keratinocytes (n = 6 embryos; total number of detected ZsGreen+ cells = 253 out of ~3000 transplanted cells). **(B)** An image showing transplanted ZsGreen+ KC-NC in the cranial mesenchyme (cm) of a 8–13 somite (som) host chick embryo immediately after injection; neural tube = nt; cm = cranial mesenchyme; som = somite. **(C)** Putative ZsGreen+ melanocytes 72 hours post injection underneath the cranial ectoderm. **(D)** HuC/D and ZsGreen double-positive neurons within the trigeminal ganglion (tgg); fb = forebrain; hb = hindbrain. **(E)** A SMA+ cranial blood vessel with a ZsGreen/SMA double positive transplanted cell. **(F)** ZsGreen/BLBP double positive glial cells (presumably Schwann cells) localized in a HuC/D-positive nerve bundle.

Next, we performed lineage tracing experiments to examine the ability of KC-NC to migrate towards stereotypical pathways *in vivo*. To this end, ZsGreen labeled KC or KC-NC that were transplanted into the head mesenchyme of 8–13 somite host chick embryos (Fig. 5B) were analyzed either 48 hours (n = 16) or 72 hours (n = 23) post-transplantation. The results showed that KC-NC were predominantly detected in locations populated by neural crest-derived cells (Fig. 5A). The majority, 60% of the KC-NC (227 cells out of 382; n = 8) localized to cranial ganglia, significantly different from only 7% of the control KC (18 cells out of 253; n = 6, p = 0.002). Similarly, 14% of the KC-NC (54 cells out of 382; n = 8) were located in the branchial arches as compared to 3% of KC controls (7 out of 253; n = 6), although the results were not statistically significant (p = 0.59). The KC-NC contributed to the full repertoire of NC derivatives, from HuC/D expressing neural (Fig. 5D) and BLBP positive glial cells (Fig. 5F), to mesenchymal αSMA positive smooth muscle lining the blood vessel walls (Fig. 5E). KC-NC also gave rise to presumptive melanoblasts below the ectoderm, although the time point for the analysis was too early to detect the melanocyte lineage marker MitF (Fig. 5C). In contrast, 75% (189 cells out of 253; n = 6 embryos) of control KC were preferentially found in the mesenchyme, corresponding to the original site of injection, whereas only 14% (52 cells out of 382; n = 8) of KC-NClocalized in the mesenchyme (p = 0.0013). These results show that KC-NC behave similarly to embryonic NC cells *in ovo* and can contribute to multiple NC derivatives, providing strong support of NC phenotype.

## Discussion

We previously found that neural crest (NC) stem cells could be derived from neonatal human epidermal keratinocytes (KC) without genetic introduction of transcription factors or reprogramming to the pluripotent state^30,38^. However, it was not clear whether NC cells could also be derived from the skin of adult donors, who are most likely in need of cellular therapies. Here we report for the first time that NC cells can be obtained from the epidermis of older adult donors ranging from 67 to 93 years of age. Adult NC cells derived from KC cultures expressed key NC markers including lineage-specific transcription factors such as SOX10, FOXD3, PAX3, the intermediate filament protein, NES and cell surface receptor, NGFR (p75NTR), similar to that observed with neonatal KC-NC^30,38^.

Previous studies showed that NC cells from different sources including gut, DRG, bone marrow, and hair bulge could be maintained in culture for short times, up to two passages^39^. Embryonic neural crest cells in traditional adherent cultures also showed limited self-renewal capacity^40,41^, which could be enhanced significantly when cells were cultured in 3D spheroids, termed crestospheres^40^. This result is in agreement with previous reports that 3D spheroid cultures enhanced the proliferation and differentiation potential of mesenchymal stem cells^42,43^, signifying the importance of cell-cell interactions in determining stem cell fate decisions^44–46^.

Although adult NC cells were derived from elderly donors, only about 10% of them were positive for senescence-associated β-galactosidase (SA β-gal), a common marker of cellular senescence^47^. In addition, they did not exhibit DNA damage as evidenced by γH2AX phosphorylation and did not upregulate cell cycle inhibitors such as P16 or P53^47,48^, but did express higher amounts of P21 as compared to neonatal NC cells. Although P21 is expressed through the p53 pathway, it can also be regulated independently^49,50^. Finally, adult KC-NC did not show statistically elevated reactive oxygen species (ROS), the product of impaired electron transport chain in mitochondria, which is known to be associated with cellular and organismal aging^48^. These results suggested that KC-NC may be epigenetically “younger” than the epidermis of the donors.

Many studies have suggested that chronological age has a significant effect on epigenetic modifications, such as DNA methylation levels^34,51–55^. In addition, mathematical algorithms have been developed to estimate the biological age of cells or tissues based on the methylation levels of sets of CpG islands. The biological age is also known as epigenetic age or DNA methylation age (DNAm age)^33,56,57^ and has been shown to be an accurate predictor of chronological age. Interestingly, application of the Horvath epigenetic algorithm as applied to 538 CpG showed that KC and KC-NC from the same donors exhibited significantly different DNAm age. While the DNAm age of KC was about the same as that of the donors (about 80 years), KC-NC were significantly “younger”, with DNAm of almost half (~40 years). Although the reason for this difference is not clear, we speculate that while KC stem cells need to proliferate frequently in order to regenerate the epidermis multiple times during a person’s lifetime, KC-NC may not be under the same evolutionary pressure, thereby “aging” at a slower pace. Alternatively, the culture conditions may have reprogrammed KC-NC to a neonatal-like state, essentially re-setting the age clock. This result suggested that despite the old age of the donors, KC-NC may have retained their multipotency *in vitro* and perhaps also *in vivo*.

Indeed, in agreement with our results with neonatal NC cells^30^, adult KC-NC could be coaxed to differentiate into functional neurons, Schwann cells, melanocytes and SMC, *in vitro*. Most notably, upon transplantation into chick embryos, KC-NC migrated along stereotypical pathways and gave rise to multiple NC derivatives, including neurons, glial cells, SMC lining the vascular wall and presumptive melanoblasts in the skin. These lineage tracing experiments in chick embryos provide strong support of the phenotype and multipotency of KC-NC. Since these cells can be derived from the human epidermis with no genetic modification or reprogramming to the pluripotent state, they have the potential to be used for treatment of neurodegenerative diseases—for which cell source remains a significant hurdle—as well as for modeling human diseases of the central or peripheral nervous system e.g. neurocristopathies^58^. Therefore, this readily accessible source of NC cells may have significant impact on regenerative medicine as well as understanding human disease and facilitating drug discovery.

## Conclusion

We showed that adult NC cells can be derived from human interfollicular KC from older donors without direct reprogramming or reprogramming to pluripotency. These cells exhibited younger epigenetic age than KC from the same advanced age donors and maintained their multipotency *in vitro* and *in vivo*. Given the accessibility of human epidermis and ease of isolation, adult KC-NC have great potential for use in stem cell therapy, disease modeling and drug discovery for treatment of neurodegenerative disorders.

## Supplementary information

supplementary figures, Figure S1, Figure S2
