# Supplementary material for: Neural crest stem cells from human epidermis of aged donors maintain their multipotency in vitro and in vivo
Source: Sci Rep. 2019 Jul 5;9:9750. doi: 10.1038/s41598-019-46140-9 (PMC6611768; doi:10.1038/s41598-019-46140-9)
Supplement: Supplementary file 1 — supplementary figures, Figure S1, Figure S2 [file 41598_2019_46140_MOESM1_ESM.pdf]

Neural crest stem cells from human epidermis of aged donors maintain their multipotency in vitro and in vivo

Running Title: Neural crest stem cells from aged skin

Samaneh Moghadasi Boroujeni[1], Alison Koontz[2], Georgios Tseropoulos[1], Laura Kerosuo[3], Pihu Mehrotra[1], Vivek K. Bajpai[4], Surya Rajan Selvam[5], Pedro Lei[1], Marianne E. Bronner[2] and Stelios T. Andreadis[1, 6, 7, §]

[1]Department of Chemical and Biological Engineering, University at Buffalo, Buffalo, NY 14260
[2] Division of Biology and Biological Engineering, California Institute of Technology, Pasadena, CA 91126
[3]NIH/NIDCR, Bethesda, MD 20892
[4] Chemical & Systems Biology, Stanford School of Medicine, Palo Alto, CA 94305
[5] Department of Psychological and Brain Sciences, University of Louisville, Kentucky, KY 40292
[6] Department of Biomedical Engineering, University at Buffalo, NY 14260
[7] Center of Excellence in Bioinformatics and Life Sciences, Buffalo, NY 14263

Supplementary Figures

Fig S1

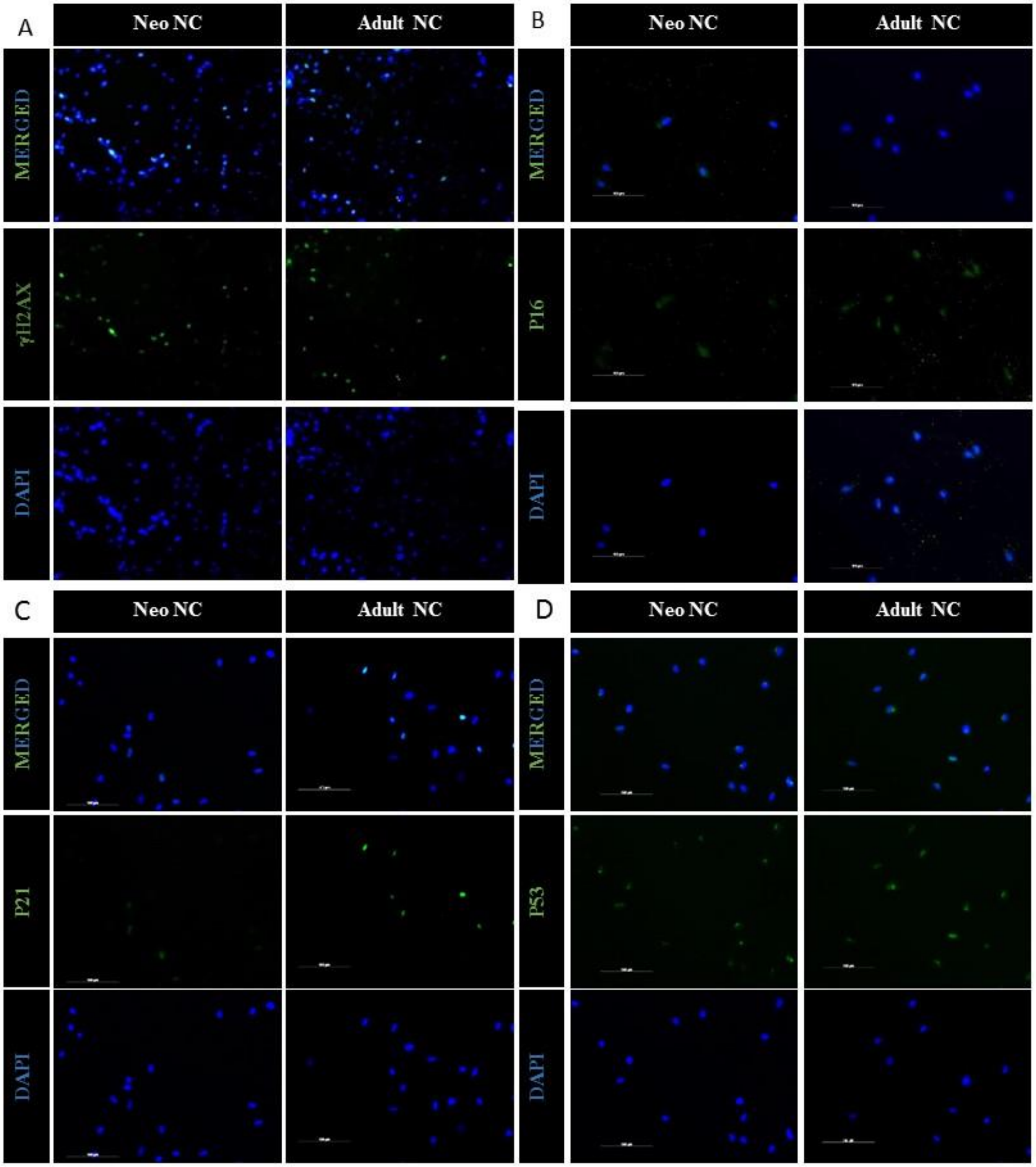

Figure S1. Immunostaining for senescence markers γH2AX, P16, P21 and P53 in adult vs. neonatal KC-NC. Scale bar is 100μm.

Fig S2

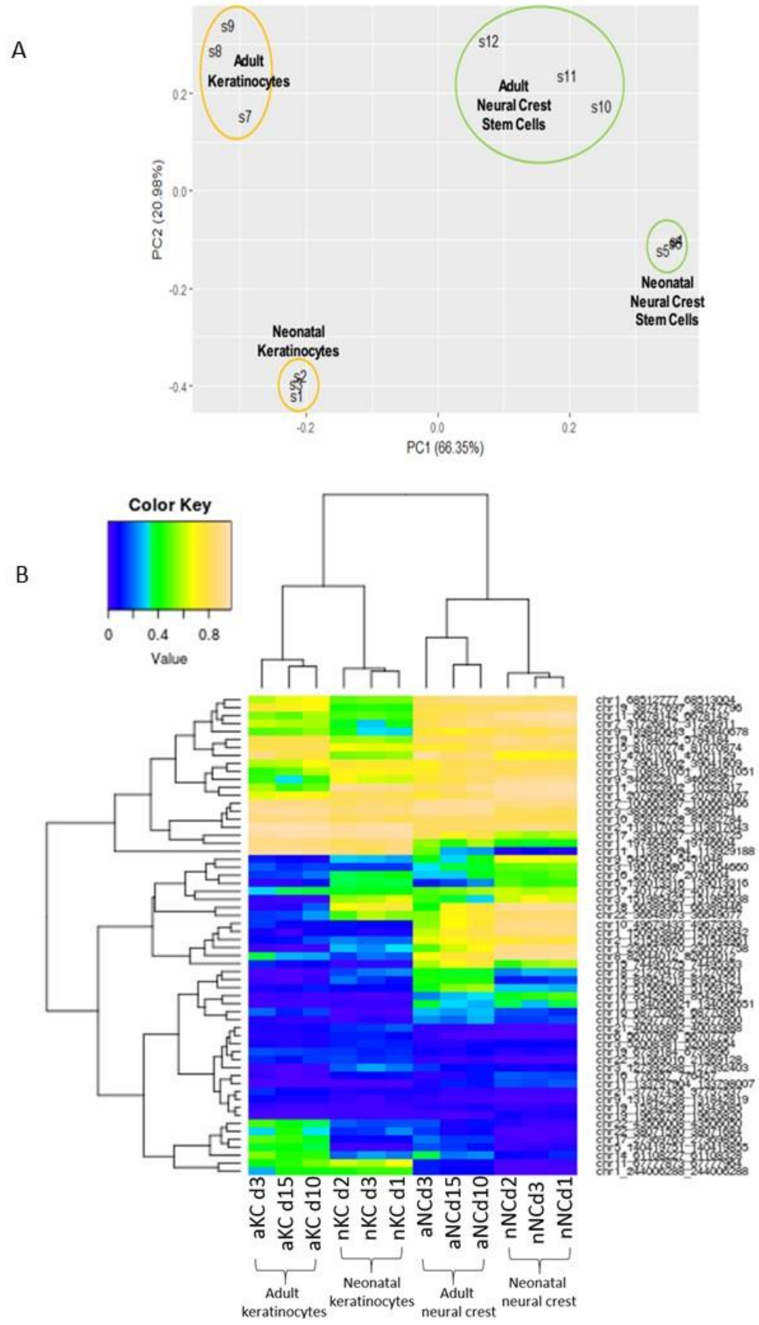

Figure S2. (A) Clustering analysis of DNA methylation from >500 age-associated gene loci in 12 human samples using multidimensional scaling. **(B)** Two-dimensional hierarchical clustering of gene loci that were differentially methylated between KC and KC-NC.
